# Evolutionary origins of polycystic ovary syndrome: An environmental mismatch disorder

**DOI:** 10.1093/emph/eoz011

**Published:** 2019-03-26

**Authors:** Mia A Charifson, Benjamin C Trumble

**Affiliations:** 1 School of Human Evolution and Social Change, Arizona State University, 900 Cady Mall, Tempe, AZ, USA; 2 Center for Evolution and Medicine, Arizona State University, 427 E. Tyler Mall, Tempe, AZ, USA

**Keywords:** polycystic ovary syndrome, evolutionary medicine, reproduction, natural selection, mismatch, infertility

## Abstract

Polycystic ovary syndrome (PCOS) is the most common female endocrine disorder and has important evolutionary implications for female reproduction and health. PCOS presents an interesting paradox, as it results in significant anovulation and potential sub-fecundity in industrialized populations, yet it has a surprisingly high prevalence and has a high heritability. In this review, we discuss an overview of PCOS, current diagnostic criteria, associated hormonal pathways and a review of proposed evolutionary hypotheses for the disorder. With a multifactorial etiology that includes ovarian function, metabolism, insulin signaling and multiple genetic risk alleles, PCOS is a complex disorder. We propose that PCOS is a mismatch between previously neutral genetic variants that evolved in physically active subsistence settings that have the potential to become harmful in sedentary industrialized environments. Sedentary obesogenic environments did not exist in ancestral times and exacerbate many of these pathways, resulting in the high prevalence and severity of PCOS today. Overall, the negative impacts of PCOS on reproductive success would likely have been minimal during most of human evolution and unlikely to generate strong selection. Future research and preventative measures should focus on these gene-environment interactions as a form of evolutionary mismatch, particularly in populations that are disproportionately affected by obesity and metabolic disorders.

**Lay Summary:**

The most severe form of polycystic ovary syndrome (PCOS) is likely a result of interactions between genetic predispositions for PCOS and modern obesogenic environments. PCOS would likely have been less severe ancestrally and the fitness reducing effects of PCOS seen today are likely a novel product of sedentary, urban environments.

## INTRODUCTION

### Polycystic ovary syndrome

Broadly, polycystic ovary syndrome (PCOS) symptoms include hyperandrogenism, anovulation and polycystic ovaries [1]; however, these symptoms are subject to wide clinical heterogeneity across patients and populations [2, 3]. In a study of couples experiencing anovulation while attending an infertility clinic in the UK, PCOS explained 90% of anovulation unrelated to pregnancy and lactation [4]. In the Dutch twin-family study, the researchers reported a high heritability (h2 = 0.72) when accounting for unique environmental influences to the prevalence of the disorder [5]. While the strong genetic heritability of PCOS could be the result of several potential factors including positive selection, balancing selection or even neutral drift to maintain gene variants associated with the disorder, the apparent lack of purifying selection against PCOS is seemingly paradoxical given high rates of anovulation [6]. Global estimates of the prevalence of PCOS range from 6 to 15%, but reach as high as 21% in the Northern Territory Aborigines of Australia [1, 7, 8]. Despite such a large frequency of the disorder, it remained undiagnosed in up to 70% of a birth cohort of females from Australia with the condition [9]. While there has been extensive clinical research into the treatment and etiology of the disorder, it remains a perplexing disease with variable consensus on what causes PCOS. In this review, we discuss an overview of PCOS, current diagnostic criteria, associated hormonal pathways and a review of evolutionary hypotheses for the disorder. Addressing PCOS through the perspective of evolutionary medicine allows us to synthesize the disparate areas of literature and propose novel evolutionary origins of PCOS and how those origins will influence its manifestation in sedentary industrial populations.

This review will critically analyse proximate mechanisms and ultimate explanations for PCOS severity and prevalence in modern populations. We will begin by:

- Reviewing the diagnostic criteria and different clinical presentations of PCOS.
- Discuss the pathophysiology and genetics of PCOS.
- Present a unified evolutionary hypothesis of PCOS.

We propose that PCOS is a mismatch between previously neutral genetic variants that evolved in physically active subsistence settings that have the potential to become harmful in sedentary industrialized environments. This summary of PCOS is an important case study of the connections between energy regulation and the female reproductive system and critically considers the clinical implications of the Western obesogenic environment on that relationship.

### Why take an evolutionary perspective on PCOS?

There are many reasons to believe an evolutionary perspective will improve the body of knowledge surrounding PCOS. An evolutionary approach to medicine asks ‘why’ do vulnerabilities to these diseases exist rather than just ‘what’ vulnerabilities exist [10–13]. Evolutionary theory can be used to probe the selective pressures that might have shaped the prevalence of PCOS and thus help to understand its root etiology. Additionally, the high prevalence and heritability of the disorder suggests there has not been strong selection against the disorder. From an evolutionary perspective, this is surprising since females with PCOS appear to have reduced fecundity. Why has a disorder like PCOS not been selected out of the population? Only positive or neutral selection on the genes involved with PCOS could explain its maintenance at such high frequencies around the world [6]. If there is positive selection for PCOS then that means related alleles may have pleiotropic effects that confer a fitness advantage in some environments or life stages. Alternatively, PCOS could be maintained by neutral selection, which would suggest the current associations between PCOS and reduced fecundity would not have existed in different environments in the human evolutionary past.

Finally, an evolutionary attempt to comprehend the high prevalence of PCOS has important implications for current and future treatment and prevention of the disorder. Modern sedentary urban environments are drastic departures from the mosaic of environments where the vast majority of human evolution took place. From an evolutionary perspective, it is imperative to ask how decreased physical activity, changes in diet, decreased pregnancies, elevated exposure to environmental pollutants in urban settings as well as decreases in immune burden have impacted the prevalence and presentation of PCOS in Western societies [14–22]. Understanding how an evolutionary mismatch in environmental conditions impacts PCOS presentation and symptoms opens new avenues of inquiry. Using this perspective, we will propose that increased exposure to an obesogenic environment might exacerbate the severity of PCOS, due to a mismatch between the evolutionary origins of PCOS and sedentary urban ecologies in which we now exist.

## CURRENT MEDICAL UNDERSTANDINGS

### Clinical diagnostic criteria

Despite the severe personal and societal burdens of PCOS, the medical community remains divided as to how PCOS should be defined and diagnosed in clinical settings. For example, the National Institutes of Health (NIH), European Society for Human Reproduction and Embryology and the Androgen Excess Society all have separate diagnostic criteria for the disorder [1, 23]. The NIH criteria from 1990 for the diagnosis of PCOS are oligo-ovulation (<6 menses/year) and clinical or biochemical hyperandrogenism [1, 23]. The European Society for Human Reproduction and Embryology/Rotterdam criteria from 2003 extends these diagnostic criteria by adding polycystic ovary morphology (PCO) (>12 follicles 2–9 mm, or ovarian volume >10 ml) to the list and requiring a PCOS diagnosis to present polycystic ovaries and at least one other criteria [1, 23]. The Androgen Excess Society criteria from 2009 has most recently suggested that hyperandrogenism (clinical and/or biochemical) be considered a necessary condition for a PCOS diagnosis along with oligo-ovulation and/or PCO; the Androgen Excess Society also encourages patients with PCOS to be regularly screened for impaired glucose tolerance and type 2 diabetes mellitus (T2DM) [1, 23, 24].

There are two main reasons why the medical community has been unable to come to consensus on the definition of PCOS. The first is a result of the normal variation in healthy females who do not have PCOS. Polycystic ovaries are found in up to 20–30% of healthy females from the UK without PCOS [25]. While PCOS explains up to 80% of clinical hyperandrogenism, 20% of females experience hyperandrogenism from other causes [23]. PCOS co-occurred with obesity in ∼30% of obese females from Spain, which was significantly more than in lean females (5%) but still means that 70% of obese females did not have PCOS [26]. Thus, it remains unclear whether obesity aggravates the symptoms of PCOS and thus increases the likelihood of diagnosis in obese females, or conversely if PCOS may predispose individuals to a greater risk of obesity [1].

The second reason why PCOS remains difficult to define and has heterogeneous diagnostic criteria is due to the unknown etiology of the disorder. A large portion of research into PCOS has been devoted to whether it is androgen production, insulin sensitivity or an alternative cause that leads to the clinical characteristics of the disorder [7, 27–29]. Unfortunately, these studies often return contradictory results, which ultimately suggest that there may be multiple causal pathways. Since there are likely multiple root causes of PCOS, this could explain why there are several phenotypic presentations of the disorder [30]. To make matters even worse, evidence shows that there may be many environmental variables such as diet, pollutants, latitude and socioeconomic status that influence the severity of the disorder [31]. A combination of varying degrees of intrinsic and extrinsic influences on the phenotype of the disorder makes conclusively establishing what ‘true’ PCOS is, nearly impossible. Most probable is that there is no single symptomology for PCOS, but several combinations of the main symptoms that can vary between individuals in cause and severity.

### PCOS phenotypes: the continuum of severity for PCOS patients

Several researchers categorize the different phenotypes of PCOS patients in a way that helps inform evolutionary hypotheses [30, 32, 33]. Based on the three main characteristics of PCOS (anovulation, hyperandrogenism and polycystic ovaries), there are four phenotypes that have been elucidated by the medical community that can still be considered variants of PCOS (Table 1). The four phenotypes consistently vary along an axis of metabolic and ovarian dysfunction from most severe (classic phenotype) to least severe (normoandrogenic PCOS) [30, 32, 33]. Type I classic PCOS (anovulation, hyperandrogenism and polycystic ovaries) is the most severe form of the disorder and patients with Type I presentation have the most acute comorbidity with insulin resistance, metabolic dysfunctions and overweight/obesity [30, 33]. Type II classic (anovulation and hyperandrogenism) is nearly as severe as Type I and shares the increased risk for metabolic dysfunction and insulin insensitivity. Ovulatory PCOS (hyperandrogenism and polycystic ovaries) lacks the characteristic reduction in ovulatory rate and menses observed in PCOS that leads to subfertility of females with PCOS, and is considered a milder form of the disorder with less severe hyperandrogenism than Type I and II as well as less severe metabolic dysfunction and higher insulin sensitivity [30, 31, 33]. The final phenotype is the normoandrogenic PCOS (anovulation and polycystic ovaries) group which is considered the mildest form of the disorder with the lowest rates of comorbid obesity, insulin resistance and other forms of metabolic dysfunction [30, 33]. While testosterone levels can be elevated slightly in normoandrogenic PCOS patients, they do not meet clinical cutoffs of hyperandrogenism [33]. Normoandrogenic PCOS patients do tend to have elevated luteinizing hormone (LH) and/or high LH/FSH ratios, which is thought to contribute to their anovulation and polycystic ovary symptoms [33].

**Table 1.** Phenotypic categories for PCOS

Phenotype	HA	PCO	A	Percentage of PCOS patients (%)^a^	Association with obesity on average?	Association with insulin resistance on average?	Effective treatment methods^b^
Type I classic	X	X	X	25.4 (range: 4.7–31.0)	Yes	Yes	Weight loss, metformin, COC, antiandrogens
Type II classic	X		X	19.3 (range: 4.7–39.7)	Yes	Yes	Weight loss, metformin, COC, antiandrogens
Ovulatory	X	X		35.3 (range: 0–72.1)	No	Yes (less than Type I & II)	Metformin, COC, antiandrogens
Normoandrogenic		X	X	20 (range: 10–32.5)	No	No	COC

There are four main PCOS phenotypes, which vary in clinical presentation, frequency, comorbidity and treatment method. Phenotypes with more clinical features and comorbidities are generally considered more severe forms of PCOS for those who experience it (i.e. Type I classic is considered the most severe phenotypic form of PCOS). It should be noted that there is considerable cross-cultural variation in the percentage of PCOS patients per phenotype. Thus, distribution of each phenotype might vary specifically by geographical region but this question that has yet to be sufficiently researched. COC, combined oral contraceptives.

a Averaged across several cross-sectional, unselected populations from the following countries Denmark, China, Australia, Mexica, Iran and Turkey [30].

b Adapted from Vrbikova and Hainer [1].

Interestingly, individuals with the more severe, classical presentation of Type I and Type II PCOS can achieve ovulatory resumption through weight loss [33]. Clinical studies observe that weight loss, as little as 5%, in obese patients with classical PCOS can ablate their symptoms (anovulation and hyperandrogenism), which suggests that some aspect of metabolism can mechanistically modulate the severity of the disorder and therefore potentially play a direct role in PCOS etiology [34, 35]. Along with weight loss, other treatment methods and their efficacy related to each phenotype reveals other proximate pathways that could cause PCOS and furthermore highlight an important message: the pathogenesis of PCOS suggests multiple root causes of the disorder.

### Comorbidity and burden of disease

Not only do the clinical presentations of the disorder vary from individual to individual but so do the impacts of PCOS, which can lead to serious and chronic complications. PCOS has a complex relationship with obesity, insulin resistance and T2DM. An estimated 30–70% of females with PCOS are also obese across a cohort of industrialized countries and a meta-analysis of females with PCOS showed that they are estimated to have a 2-fold increased incidence of impaired glucose tolerance and T2DM [1, 24]. Insulin insensitivity was observed in ∼40% of cohort PCOS patients from the USA and was observed independent of obesity in some patients as well [36]. Furthermore, patients with PCOS are at an increased risk of experiencing T2DM, stroke and cardiovascular disease, which are all included in the top 10 global leading causes of disability among females [37, 38]. Individuals with PCOS report an overall lower health-related quality of life, displaying increased rates of depression, anxiety, self-harm and suicidal behavior [37]. PCOS can result in hyperandrogenism, hirsutism (excess facial hair growth) as well as acne, anovulation, irregular menstrual cycles and muscular hypertrophy [7]. Anovulation related to PCOS accounts for ∼27% of infertility in industrialized populations and up to 72% of infertility in some study populations [7, 39]. A longitudinal Finnish study of PCOS patients found that they have reduced fertility overall with significantly smaller family sizes (7.7% with 3 or more live births compared to 16.8%) [40]. However, PCOS-related anovulation is intermittent and results in sub-fecundity, not complete infertility; a longitudinal Swedish study found females with PCOS had equivalent likelihood of having at least one child compared to controls (87% to 92% respectively) [39, 41]. In the USA, the direct costs of PCOS are ∼$4.36 billion per year, which do not include the indirect costs of PCOS-related infertility treatment, or the increased probability of developing T2DM [37]. An estimated 70% of people with PCOS are undiagnosed meaning that the financial, personal and societal burden of this disorder are even higher than currently understood [9].

### Treatments vary in efficacy for different PCOS phenotypes

Weight loss, insulin sensitizers, oral contraceptives and antiandrogens are the most common treatment methods for patients with PCOS, though each method varies in efficacy in which aspect of the disorder they are able to treat [1, 7, 23]. Weight loss is an effective strategy in treating anovulation and menstrual irregularity in females with PCOS and many researchers conclude that these improvements are related to reductions in elevated blood insulin levels (hyperinsulinemia) and improvements in insulin sensitivity [1, 34, 35, 42]; insulin resistance is considered, by many, a casual pathway in the development of PCOS [28, 43, 44]. Evidence that insulin mediates the effects of weight loss on PCOS severity is also seen through the use of insulin sensitizers to treat PCOS. Insulin-sensitizing drugs notably improve ovulation and insulin sensitivity in both obese and non-obese PCOS patients, though they do not contribute to weight loss [1]. Given that insulin-sensitizers are the most reliable form of improving metabolic profiles in PCOS patients, they may be the best line of treatment for PCOS patients with insulin resistance, especially if combined with some other weight loss strategy in obese patients. Unfortunately, many patients with PCOS have underlying genetic predispositions for obesity and related reduced insulin sensitivity, which can make weight loss difficult [7, 23, 45]. Both exercise and low glycemic index diets have been shown to clinically reduce the severity of PCOS among patients who adopt either as a therapeutic treatment [1, 42, 46, 47]. Since exercise and dietary improvements also are known to reduce insulin levels in the blood as well as improve existing insulin insensitivity, their efficacy in treating PCOS is further evidence that affecting insulin pathways can have direct effects on PCOS pathophysiology [1, 42, 46, 47]. The effect of weight loss in lean patients with PCOS who are not overweight or obese in order to restore ovulatory function has not been studied sufficiently, which leaves an important question as to whether or not affecting insulin pathways in the absence of obesity will alleviate PCOS symptoms [39].

Other treatment options for PCOS do not rely on insulin dependent pathways, but treat the dysregulated ovarian hormonal milieu in PCOS patients. Combined oral contraceptives (usually a mixture of progesterone and estrogen based hormonal contraceptives) are effective methods to treat hormonal irregularity in females with PCOS [1, 7]. In obese patients, oral contraceptives have diminished efficacy and some evidence suggests that high dose oral contraceptives might possibly further reduce insulin sensitivity in all patients [1, 42]. Since obese PCOS patients are more likely to have higher degrees of insulin insensitivity, affecting only hypothalamic-gonadal axis (HPG) axis dysregulation may not address the ability for insulin pathways to contribute to PCOS pathogenesis. This could explain the lowered efficacy of oral contraceptives in obese PCOS patients. Furthermore, there is great cause for concern of the use of oral contraceptives in obese PCOS patients as it is common knowledge that estrogens increase the risk of blood clotting, which in obese patients may increase the risk of coronary heart disease [1, 42]. Antiandrogens are effective in treating hirsutism and sometimes acne in most PCOS patients for whom that is of serious concern, but are not effective in addressing anovulation, menstrual irregularity and metabolic dysfunction, and being such, they are often used in conjunction with oral contraceptives [7, 48]. The fact that insulin sensitizers, and not antiandrogens, are the more effective treatment of PCOS suggests that insulin sensitivity might be at the core of the disorder; though it is important to note that not all patients have reduced insulin sensitivity. Moving away from clinical cutoffs of ‘insulin resistance’, and instead considering reduced insulin sensitivity on a continuum might better inform this question. Next, we will build upon these insights from clinical understandings of PCOS to incorporate both systems biology and genetics research on the disorder to better examine its origins within the body as well as in evolutionary time.

## UNDERSTANDING THE PROXIMATE PATHWAYS INVOLVED IN PCOS

### Pathways to dysregulation

**Figure 1.**
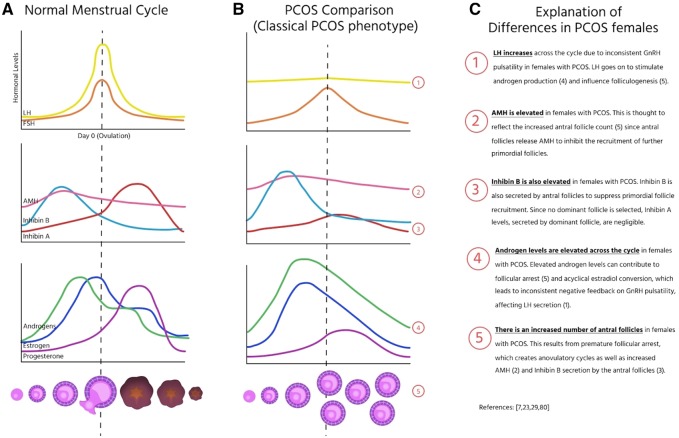
Hormonal differences between PCOS and non-PCOS individuals. The hormonal profiles throughout folliculogenesis and ovulation in (A) individuals without PCOS on average to compared to (B) individuals with Type I classic PCOS phenotype and (C) a description of the relevant differences and how they affect these processes

While there is a high degree of heterogeneity in symptoms of PCOS, there are several consistent markers. Common symptoms include elevated androgens, elevated gonadotropin releasing-hormone (GnRH) which results in an elevated frequency and amplitude of LH and follicle stimulating hormone (FSH), and reduced sex-hormone binding globulin (SHBG), as well as acyclical estrogen levels [2, 7, 48]. Elevated androgen production leads to non-cyclical conversion of androgens to estrogens which provide irregular negative feedback to the hypothalamus, resulting in elevated levels of LH and low or normal levels of FSH. The high LH/FSH ratio further promotes elevated androgen levels, as LH stimulates the production of androgens from theca cells, but furthermore can lead to the arrest of follicular development in tangent with elevated androgens [7]. Taken together these markers suggest that PCOS is the result of deregulated ovarian function in a continuous feedback loop with no clear beginning or end (Fig. 1).

### Genetic polymorphisms attributed to PCOS pathogenesis

There are a number of genetic polymorphisms that have been associated with PCOS and point to the importance of hyperandrogenism and insulin sensitivity as potential causal mechanisms in PCOS etiology. There is no strong evidence of positive selection on any genes (individual single nucleotide polymorphisms or polygenic selection) related to PCOS [45, 49]. Not only is there absence of a strong genetic signature of selection on PCOS, but one study concluded that there was evidence for genetic drift, which established frequencies of genetic polymorphisms related to PCOS into five distinct haplotypes that mapped to distinct geographic regions [49]. Therefore, even though 72% of susceptibility to PCOS was explained by genetic contributions in one environmental context, that contribution appears to be the result of by many interrelated as well as unrelated genes (Table 2) [5, 23, 50]. The genetic variants linked to PCOS susceptibility range from metabolic markers of disease risk to genes with a direct role in the HPG axis, reaffirming the hypothesis that PCOS results from several possible genetic predispositions that affect a number of biological processes whose additive effect can lead to PCOS pathophysiology [7, 23, 45, 50]. Further understanding the pathways affected by these genetic variants is necessary to evaluate why the possibly fitness reducing effects of PCOS were not selected against in ancestral times, but instead underwent either genetic drift or balancing selection.

**Table 2.** Genetic polymorphisms and associated pathways implicated in PCOS

Proposed function in PCOS	Gene variant associated with PCOS	References
Obesity	*FTO, ADIPOQ*	[7]
Insulin pathways	*INSR, IRS1, IRS2, PPARG, CAPN10*	[7]
Delayed menopause	*RAD50, EGFR*	[7]
Hyperandrogenism	*CYP1A1, CYP11A, CYP17A1, CYP19, HSD17B6*	[7]
	*DENNID1A, RAB5B, LHCGR, INSR*	[50]
Anovulation	*FSHR, LHCGR, AMHR2*	[7]
	*FSHB, EGFR*	[45]
Inflammation	*ILA1, IL1B, IL6, IL18, FBN3, TNF, MEP1A*	[7]

There have been a wide range of genetic polymorphisms associated with PCOS and their proposed physiological contribution to PCOS pathophysiology vary from metabolic, reproductive and immune pathways.

Elevated androgen levels are the most notable HPG disruption in patients with PCOS. Clinical research suggests that hyperandrogenism precedes both anovulation and menstrual irregularities [7, 27]. There are multiple pathways through which androgen levels can be elevated, and several androgen related disorders are comorbid with PCOS, suggesting multiple potential mechanisms that could result in PCOS [23, 51]. For example, non-classical adrenal hyperplasia can result in elevated androgens, and one meta-analysis reported 20–30% of patients with PCOS have adrenal androgen excess [7, 51, 52]. Genetic predispositions could also explain hyperandrogenism in some PCOS patients based on research showing two candidate genes, *DENNIDA* and *CYP71A*, related to androgen synthesis that could result in excessive levels of free androgens in PCOS patients [7, 53]. Because insulin pathways can contribute to androgen levels in the body, antiandrogens treatments have inconsistent efficacy in resolving metabolic and menstrual dysfunction when not addressing predispositions for insulin insensitivity simultaneously [1, 7]. In fact, hyperinsulinemia in and of itself can be responsible for hyperandrogenism since insulin and Insulin-like growth factor 1 (IGF-1) can act as co-gonadotropins, stimulating ovarian steroid production along with gonadotropins [28, 48]. Elevated levels of anti-Mullerian hormone might have a synergistic relationship with hyperandrogenism; evidence from rodent models indicates that excess prenatal exposure to AMH or androgens can result in PCOS-like phenotypes [7, 23, 29, 54]. Therefore, both genetic predispositions for hyperandrogenism and prenatal androgen exposure, an environmental influence, could contribute to PCOS pathophysiology.

In addition to the possible genetic pathways associated with hyperandrogenism, genetic predispositions for obesity, insulin resistance and T2DM may help explain disruptions of insulin pathways that can result in PCOS. Risk alleles like *FTO, ADIPOQ, INSR, IRS1, IRS2, PPARG, CAPN10* may leave individuals more vulnerable to insulin resistance and/or adiposity (which can result in peripheral insulin resistance), leading to high circulating insulin [1, 7, 23, 28, 45]. High circulating insulin may then trigger hyperinsulinemia in the ovaries, where insulin sensitivity remains normal despite peripheral insensitivity [48, 55]. Additionally, ∼50% of females with PCOS have a defect in the insulin-pathway downstream of the receptor in the ovaries that may contribute to PCOS [28].

Treatment of Type I diabetes can also contribute to some cases of PCOS. Individuals with Type I diabetes are unable to produce insulin themselves and thus are treated with exogenous injections of insulin on an almost daily basis [56, 57]. These injections of insulin can result in hyperinsulinemia and influence ovarian steroidogenesis in the same way hyperinsulinemia as a result of obesity might [56]. Consistent with this hypothesis, a cohort of female patients from Spain with Type I diabetes are at an increased risk of PCOS with 39% of females with Type I diabetes having comorbid PCOS [57]. The parallel drawn between Type I and Type II diabetes in the pathophysiology of PCOS makes a strong argument that environmental or genetic features that increase insulin in the bloodstream will contribute to PCOS severity.

There is evidence that some weight gain occurring at the outset of PCOS symptomology could be due to hyperandrogenism, which has been linked to higher central fat deposition in females [1]. However, predispositions for obesity are the result of complex genetic and environmental interactions [58]. There is no reason to believe that hyperandrogenism is sufficient to explain all of obesity in PCOS patients given these complex interactions; however, it is interesting to consider how the outset of PCOS might in some ways trigger these underlying gene-environment predispositions for obesity.

**Figure 2.**
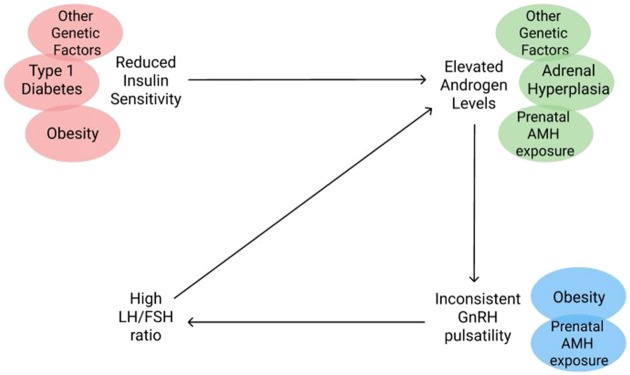
The intersection of PCOS comorbidities in PCOS pathophysiology. Comorbidity in patients with PCOS and where along the pathophysiology of PCOS each comorbidity may play a relevant role in the pathogenesis of the disorder

Taken together, these myriad genetic predispositions for either insulin resistance or hyperandrogenism suggest that PCOS is likely not the result of a singular selection target nor a singular group of selection targets but rather is the result several independent genetic changes, many of which may have pleiotropic effects (Fig. 2). By understanding the possible proximate mechanisms of PCOS we are able to conclude that PCOS was unlikely to be the result of selecting for a single genotype but rather several. Furthermore, there is evidence that these genetic predispositions can be induced by environmental triggers (i.e. prenatal androgen exposure, obesity-related hyperinsulinemia and/or insulin injections). We propose that the spectrum of PCOS severity seen today might result from an interaction between gene variants related to PCOS and aforementioned environmental triggers in some individuals with PCOS.

## GENERATING ULTIMATE EVOLUTIONARY HYPOTHESES FOR THE ORIGINS OF PCOS

With an understanding that both genetic and environmental disruptions can influence the HPG and insulin pathways that can result in PCOS, there are many important questions to ask. First, we evaluate if environment differences in ancestral environments and modern sedentary urban environments could create differences in PCOS phenotype distribution. Then, we hypothesize regarding whether there were selective pressures for or against the ancestral PCOS phenotypes in ancestral ecologies. Finally, we will discuss what conclusions can be made about the evolutionary origins of PCOS.

PCOS likely emerged within an ancestral environment that occurred prior to the advent of agriculture. We see PCOS in rates of at least 6% in nearly all ethnicities (excluding East Asians) [59, 60]. It is unlikely that a trait such as PCOS arose independently in every region of the world, unless it had a very large fitness advantage [6, 49, 61]. Since it exists at high frequencies across populations (6–21%), but not as high as traits with convergent evolution (i.e. lactase persistence), that is not likely the case [62]. Therefore, it was likely a derived trait that existed within humans before the dispersal out of Africa [61].

### Would the PCOS phenotype be different in ancestral environments?

In ancestral environments, obesity was rare and the influence of extrinsic metabolic disruption (i.e. urban sedentary life, high fat/high carbohydrate diet) would have been much lower, probably resulting in low rates of the severe PCOS phenotype [63, 64]. Among extant hunter-gatherers, as well as forager horticulturalists, obesity and related metabolic disruptions are relatively rare, as are metabolic-related reproductive conditions [18, 63–65]. Within the USA, however, obesity affects nearly two-thirds of Americans with rates increasing throughout the decade [58]. The mismatch between current and ancestral conditions, including differences in diet, and physical activity, all impact insulin pathways in sedentary urban environments, resulting in higher rates of metabolic disease and potentially higher rates of PCOS [14–17, 21, 22, 63, 66, 67]. This mismatch between current obesogenic environments and its effect on the severity of PCOS has been suggested by several other researchers as well [22, 31, 43]. Other changes such as immune burden also impact overall calorie balance and metabolic function [21, 63]. While there is no data on PCOS in ancestral environments, we predict that obesity-related classical PCOS phenotypes were likely uncommon, and thus the relatively rare lean PCOS phenotype is likely how PCOS could have presented ancestrally, if it did at all.

### How would ecology have shaped the evolution of PCOS in those ancestral environments?

Natural selection can function through affecting two main variables: reproductive success and mortality to the extent that it impacts reproductive success. The observation that the most severe forms of the PCOS phenotype reduce fecundity suggests it would have important implications for natural selection. Some researchers have suggested the reduced fecundity could be balanced out by the possibility that PCOS improves the probability of surviving in ancestral environments by allowing for more robust bodies as a result of hyperandrogenism [7, 39, 68]. PCOS and hyperandrogenism have both been associated with higher bone mass density compared to controls [68]. The effect of hyperandrogenism on bone mass density appears to be greatest in individuals with higher menstrual regularity [68]. Thus, it is possible that ancestral females with PCOS did indeed have higher bone mass density than other females; however, there is little research looking into associations between reproduction, bone mass density and survivorship in ancestral environments [69–71]. In sedentary industrial populations, there is an association between low bone mass density and survivorship, particularly for older individuals [72]. From an evolutionary perspective, survival is only important in so far as it ensures individuals survive to reproduce [11]. Therefore, the force of selection on elderly bone mass density and survival at late ages would have been weak. Since there is limited evidence therefore that PCOS is adaptive in terms of survival, what are the implications of PCOS when it comes to reproductive success?

From an evolutionary perspective, PCOS is perplexing because it confers lower overall fecundity and fertility today, which decreases the reproductive success of individuals, and thus would be selected against [7, 39]. However, there are several reasons to believe that there was not strong selection for or against PCOS in ancestral environments. First, it is likely that a less severe presentation of the disorder was most common in ancestral environments, given the relative scarcity of metabolic disease [30, 33]. This would mean that there is a higher probability of ancestral PCOS females cycling and less of a fecundity difference between them and non-PCOS females. Second, there would less of a difference in overall fertility between non-PCOS and PCOS females in ancestral environments. Females in ancestral environments spent much of their reproductive careers pregnant or lactating, and thus have only one quarter the number of menstrual cycles as females living in industrial populations [19, 73, 74]. This is a result of high fertility, nutritional stress, immune activation and long breastfeeding-periods [74]. Therefore, ancestral PCOS females would have likely had a higher rate of ovulation than PCOS females today and its apparent effect on reproductive success would have been lessened by the increased parity of all ancestral females. In modern times, the decreased parity of females in post-demographic transition societies makes both proportional parity differences between PCOS and non-PCOS females greater and the fecundity differences more apparent [18, 22, 75]. As ancestral PCOS is hypothesized to be rare, or of a milder phenotype, there would be few negative impacts of that condition. Thus these previously neutral genetic variants in active subsistence settings only become harmful in sedentary industrialized environments and are an example of a mismatch condition. Obesogenic sedentary environments were rare or non-existent in ancestral times, and their existence today exacerbates many of these PCOS-related metabolic pathways, resulting in the high prevalence and severity of PCOS today. Given these arguments, the impact of PCOS on reproductive success would be much smaller ancestrally and unlikely to generate a strong selective force.

**Figure 3.**
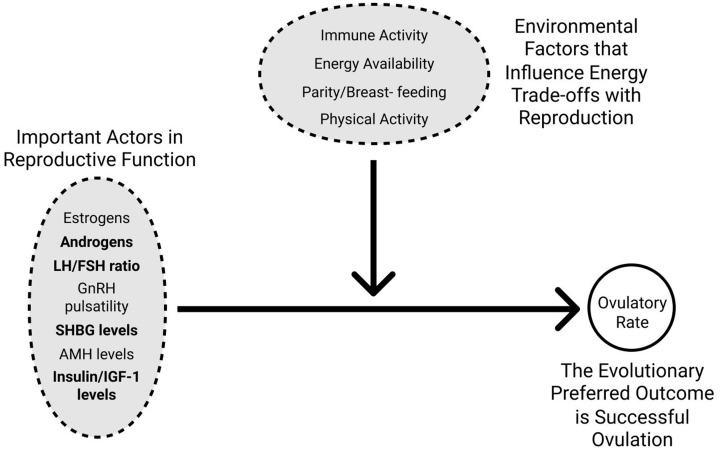
Proposed interaction between PCOS genetic susceptibility and environmental factors on ovulation rates. Successful ovulation is influenced by both the energy available to be used in reproductive function and genetic factors that affect the important actors in the female reproductive axis. Energy available for reproductive function for the purpose of ovulation will be traded off with immune activity, physical activity, parity and breastfeeding. The evolutionarily desirable outcome of a high ovulatory rate will therefore be a result of both environmental factors and the internal actors on reproductive function. Bolded actors in reproductive function are those known to have gene variants that are associated with PCOS

Our prediction that anovulation in PCOS females today is in part a mismatch between neutral gene variants and modern urban, sedentary environments requires important consideration. We hypothesize that anovulation in individuals with PCOS could be greater today than in ancestral environments due to a mismatch with current states of energy excess. Ovulation is achieved as the result of several biological actors within the female reproductive axis (i.e. GnRH pulsatility, sex steroid ratios and insulin/IGF-1 levels). However, the female reproductive system requires energy, which must be traded off between other biological processes within the body including physical activity, immune burden and breastfeeding [18, 76–79] (Fig. 3). In ancestral ecologies, despite some individuals possessing genetic variants associated with PCOS that likely influenced a number of internal mechanisms necessary for successful ovulation, the more important limiting factor for ovulatory rates was most likely the trade-offs they experienced between ovarian function and high immune burden, high levels of physical activity, high parity, prolonged periods of breastfeeding and in some cases, seasonal variations in food availability [18, 76–79]. In modern urban, sedentary ecologies, individuals with PCOS not only face the genetic variants that have persisted since ancestral times, but also have excessive energy that could be devoted to exacerbating the imbalanced ovarian steroidogenesis and insulin/IGF-1 levels. The loosening of energetic constraints on ovulation likely created a more apparent difference in ovulatory rates between those individuals who did and did not possess PCOS gene variants. The additional metabolic disturbances, resultant of urban, sedentary ecologies, may further interact with these PCOS gene variants to produce the more severe classic PCOS phenotype, which serves to intensify this difference in ovulatory rates.

In summary, we hypothesize that in ancestral ecologies, differences in ovulatory rates between females with and without PCOS genotypes were likely negligible due to high rates of parity and lactation [18, 19, 80, 81]. A more severe PCOS phenotype produced by urban, sedentary environments today increases anovulation in modern females with PCOS, thus creating a novel fitness reducing effect of PCOS that was unlikely to produce selective pressures in ancestral environments. This explanation accounts for why PCOS genotypes display no signals of negative or positive selection, despite its apparent fitness reducing effects today.

### What are possible adaptive explanations for PCOS in ancestral environments?

Is neutral theory enough to explain PCOS frequency in modern populations? Some researchers believe that genetic drift is not an adequate explanation and instead, adaptive explanations for PCOS need to be explored because a genetic history of random drift cannot explain its high frequency across geographic regions, especially considering its possibly fitness reducing effects [29, 39, 45, 61, 82]. This line of research is particularly motivated by genetic research on PCOS concluding that there is a history of balancing selection that explains its geographic patterning and modern frequencies [45, 49]. A few compelling adaptive hypotheses have been proposed including faster resumption of menses, obligate ovulation in times of energy scarcity, transgenerational effects of prenatal androgen exposure and delayed menopause [39, 61, 82, 83]. The resumption of menses following lactational amenorrhea is preceded by a rapid increase in insulin levels due to peripheral insulin resistance that is thought to facilitate the resumption of ovarian function to pre-pregnancy levels [84]. Similar mechanisms might be involved with the timing of menarche during puberty [84]. Therefore, elevated insulin levels as a result of insulin insensitivity could be adaptive if both earlier puberty and shorter lactational amenorrhea increase reproductive success [39, 83]. Alternatively, the relative insulin insensitivity of PCOS females compared to non-PCOS females is proposed to be adaptive in contexts of energy scarcity when non-PCOS females have been shown to downregulate their ovarian function [39, 77, 83]. Building off this hypothesis, one review posited that this phenotype would have been adaptive and selected for during the Neolithic revolution, a period of high energy scarcity and variability [39, 43]. If PCOS phenotypes procured adaptive benefits in times of energy scarcity, then one can conclude that PCOS phenotypes in modern environments of high energy availability is another form of mismatch [39]. However, unlike our mismatch hypothesis, these possibly facultative aspects of PCOS phenotypes suggest that there are specific environmental conditions in which the gene variants associated with PCOS will become adaptive for reproductive success. Yet, evidence to corroborate adaptive benefits such as this in certain environmental contexts has yet to be shown among PCOS females [39]. To the contrary, a mismatch model that assumes neutral drift for PCOS gene variants suggests that it was never adaptive but there are environmental conditions today which modulate the severity of the phenotypic presentation of those gene variants.

Another adaptive hypotheses for the PCOS phenotype in humans draws upon comparative species analysis that points to a role of developmental androgen exposure in developing a PCOS-like phenotype [29, 83]. Evidence also shows that *in utero* exposure to androgens in some rodent species, sheep and rhesus macaques animal models can produce a PCOS-like phenotype [29, 83]. Non-human research, namely done in rodents, has revealed that neonatal androgenization, in response to maternal stress, impacts the effects of estrogen on sexual receptivity and behavior [85, 86]. Furthermore, high prenatal androgen exposure can create a high androgenic state, which may be associated with a metabolic profile favorable for energy scarcity, therefore preparing offspring for an environment with high resource variability [83]. This hypothesis invokes a gene-environment interaction between PCOS gene variants and high prenatal androgen exposure to prepare *in utero* for a possibly volatile environment rather than a mismatch between environment and genotype. This line of inquiry has been less studied than other hypotheses, and creates several lines of yet unanswered questions. What effect do the gene variants have in the absence of high prenatal androgen exposure? Do the gene variants themselves create a prenatal environment with high androgen exposure? If so, it would not be a facultative response to maternal stress while *in utero*. Is maternal stress a reliable signal of resource variability in later life? Ultimately, this hypothesis leads to a chicken-and-egg dilemma that is difficult to disentangle but worthy of further research into comparative species analysis.

Finally, research investigating the genetic signatures of PCOS suggest that there has been no recent positive selection of PCOS-related genes and evoke hypotheses of balancing selection to maintain its high frequencies across populations [45]. We have already discussed one possibility of balancing selection between bone density and PCOS, which would hypothetically be balanced by increasing survival despite lowering fecundity [39, 61, 68]. However, it has also been suggested that genetic predispositions for PCOS create balancing selection through their negative effects on fecundity and positive effects of slower ovarian aging [45]. Similarly, others have also suggested that PCOS could have been positively selected for the role it plays in delaying menopause [29, 39, 87–89]. However, the potentially ‘protective’ aspects of PCOS against ovarian aging are not seen in females with PCOS who have regular menstruation [88]. In other words, these effects are not present in the less severe phenotypes of the disorder, which would likely have been the ancestrally common form of PCOS, if PCOS occurred in ancestral environments. Therefore, it seems unlikely that there would have been positive selection on PCOS in delaying ovarian aging.

Unfortunately, many of the evolutionary hypotheses about PCOS have remained just that: hypotheses. While the literature reviewed here suggests that many adaptive hypotheses seem unconvincing, it would still be prudent to test these hypotheses. The most relevant course of action is to investigate the PCOS spectrum in subsistence populations who live in ecologies and lifestyles that most closely resemble the experiences of our ancestors. While evolutionary origin stories are hard to prove, it is possible to test the functional significance of the lean PCOS phenotype on reproductive success. For example, if hyperandrogenism due to PCOS is a functional adaptation to increase bone mineral density, then treating hyperandrogenism in PCOS patients could be placed at higher risk of osteoporosis later in life.

Based on the current state of the literature in regards to evolutionary hypotheses of PCOS, it seems more likely that PCOS is the result of a number of diverse, potentially pleiotropic gene interactions that all can result in a similar disease phenotype in some environments [45, 53]. These diverse genetic changes have been linked to obesity, insulin resistance, SHBG and epidermal growth factor receptors [45]. The variation in mechanism of action across these pathways may help explain the heterogeneity in PCOS symptoms we see today. Arguments for positive selection on PCOS currently lack sufficient evidence, and a more plausible explanation is that that there was no selection for or against PCOS, and that the syndrome only presents in its most severe form in certain gene-environment interactions that would not have occurred in ancestral environments. These alleles disrupt either the HPG and/or insulin pathways in directions that produce a continuum of PCOS phenotypes that are exacerbated in obesogenic sedentary environments—a form of evolutionary mismatch.

## CONCLUSIONS

Taking an evolutionary perspective of PCOS gains insights and also creates new lines of research for the diagnosis and treatment of PCOS. In this review, we first critically examined diagnostic criteria and clinical presentations of PCOS. Previous statements by PCOS taskforces have stated that insulin resistance and metabolic dysfunction are not aspects of health that need to be considered in the diagnosis or treatment of PCOS [51]. Following a discussion of research on the pathophysiology and genetics of PCOS, we conclude that ample literature in the past decade disagrees with those statements [28, 30, 33, 39, 44, 55, 90]. It is clear that insulin plays a crucial role in the pathophysiology of PCOS and can also intersect with other root causes to generate different phenotypes of varying severity. Therefore, insulin sensitivity it is not only important for diagnosing PCOS because it provides insight into the severity of the disorder, but it also plays a crucial role in treatment. When the role of insulin resistance in PCOS is overlooked, possible side effects and targets for treatment will go unaddressed. Additionally, we examined literature suggesting both environmental and genetic factors that disrupt HPG and/or insulin pathways can modulate the severity of the disorder. Unifying our review of the proximate pathways of PCOS with evolutionary theory, we concluded that PCOS was likely not under selection in ancestral times. Because the disorder was likely only experienced in its mildest form due to differences in ancestral ecologies and lifestyle, the fitness reducing effects of the disorder that are seen today were likely negligible prior to the advent of sedentary and urban modern environments. Thus, PCOS is a mismatch between current environments and genetic variants that were likely harmless in ancestral times.

This understanding of PCOS as a mismatch disorder raises concerns for populations that are transitioning to a more sedentary industrial lifestyle marked by higher caloric intake and low levels of physical activity [22]. As extant subsistence populations become more acculturated obesity and diabetes are likely to grow in prevalence, which will exacerbate existing predispositions for PCOS [8, 91–93]. The highest of rates of PCOS (21% of reproductive age females) are seen in the Northern Australian Aborigines, a population that has already undergone this transition [8]. Furthermore, these concerns also extend to socioeconomic disparities that can manifest as health disparities. Across developed nations, communities with lower socioeconomic status experience obesity and diabetes at higher rates [22, 38, 91]. These populations are also at higher risk of the more severe forms of PCOS that include a higher chronic burden across the reproductive lifespan. If the mismatch between genetic predispositions for PCOS and urban, sedentary and industrialized lifestyle is not acknowledged, populations who are already marginalized by lower socioeconomic backgrounds, weakened healthcare infrastructure and possibly limited governmental support, will likely suffer from PCOS not only more so than other populations but also silently [94–96]. By taking a proactive approach now to inform medical professionals serving those communities on these gene-environment interactions, they might be more equipped to recognize and treat the signs and symptoms of PCOS early on.

Finally, this review reveals several promising avenues for future research to propel the field of PCOS research forward. If interested in confirming hypotheses of positive selection for PCOS, it would be helpful to test whether or not females with PCOS that are able to conceive resume menses faster than non-PCOS females, or have shorter inter-birth intervals. Furthermore, one could test whether or not females with ovulatory PCOS differ in their response to extreme exercise compared to non-PCOS females who would likely become amenorrhoeic in times of extreme exercise. While it is generally impossible to truly test hypotheses about positive selective advantages in ancestral populations that no longer exist, there is the opportunity to study the prevalence and phenotype of the disorder among extant subsistence/hunter-gatherer populations. These populations fall short of representing the entire mosaic of ancestral ecologies, but are likely the best proxy for ancestral environments and lifestyles. Research among these subsistence populations could test to see if sub-clinical insulin sensitivity occurs and accrues benefits for reproduction, which could confirm evolutionary hypotheses of adaptive benefits for PCOS in non-obesogenic environments. Finally, phylogenetic analyses are an open avenue to assess hypotheses of positive selection for PCOS gene variants. Comparative research that might point to a conserved mechanism across species could highlight a facultative role of variants associated with PCOS in survival and development and should be a high priority in research when considering the evolutionary origins of PCOS.

Despite the heavy burden, high prevalence and extensive research, the roles of environment and lifestyle in the etiology of PCOS remain poorly understood. This review highlights how evolutionary perspectives can be useful for investigating links between ecology and PCOS and motivate future research to better understand it, in order to ultimately ease the burden of those affected.
